# Incidental/Misdiagnosed Intrahepatic Cholangiocarcinoma and Combined Hepatocellular-Cholangiocarcinoma in Patients Undergoing Living Donor Liver Transplantation

**DOI:** 10.3390/jcm15187206

**Published:** 2026-09-17

**Authors:** Tonguç Utku Yilmaz, Ali Özer, Murat Yildar, Hikmet Aktaş, Bakır Bati, Meltem Güner Can, Bedriye Koyuncu Sökmen, Hale Kirimlioğlu, Hamdi Karakayali

**Affiliations:** 1 Department of General Surgery, School of Medicine, Acıbadem Mehmet Ali Aydınlar University, Istanbul 34303, Türkiye; 2 Organ Transplantation Unit, Atakent Hospital, Acibadem Mehmet Ali Aydinlar University, Istanbul 34303, Türkiye; 3 Organ Transplantation Unit, Acibadem Bursa Hospital, Bursa 16110, Türkiye; 4 Department of Anesthesiology and Reanimation, Acibadem Mehmet Ali Aydinlar University, Istanbul 34752, Türkiye; 5 Department of Radiology, Atakent Hospital, Acibadem Mehmet Ali Aydinlar University, Istanbul 34303, Türkiye; 6 Department of Pathology, Acibadem Mehmet Ali Aydinlar University, Istanbul 34684, Türkiye

**Keywords:** cholangiocarcinoma, hepatocellular carcinoma, living donor liver transplantation, incidental, recurrence

## Abstract

**Background/Objectives**: Mixed hepatocellular-cholangiocarcinoma and intrahepatic cholangiocarcinoma (CCA) are increasingly reported in patients with cirrhosis. Traditionally, these tumor types have not been considered indications for liver transplantation. We aimed to present our single-center experience for incidental CCA following liver transplantation. **Methods**: Among the 985 living donor liver transplantations, 261 cases were initially diagnosed as hepatocellular carcinoma. We retrospectively reviewed demographic data, preoperative radiological findings, pathology results, and postoperative outcomes. We classified tumors according to the Goodman classification. **Results**: Cholangiocarcinoma was identified in the explant pathology of 18 patients. According to the Goodman classification, five patients (27%) had type 1 tumors; eight (44.4%) had type 2 tumors; and five (27.8%) had sole CCA lesions. Tumor recurrence occurred in six patients (33.3%). The mean survival time was 61.6 ± 12 months. The mean disease-free survival was 10.67 ± 5.7 months. Disease-free survival times for Goodman type I, type II, and sole cholangiocarcinoma were 14.00 ± 5.57, 8 ± 5.66, and 6.00 ± 0.00 months, respectively. The 1-year and 5-year survival rates were 88% and 55%, respectively. Four patients died due to CCA; the remaining deaths were due to sepsis, COVID-19, amyotrophic lateral sclerosis, and lung squamous cell carcinoma. **Conclusions**: The misdiagnosed/incidental cholangiocarcinoma in transplanted patients with cirrhosis is rarely seen with high recurrence rates. Although transplant oncology is gaining prominence, preoperative evaluation and postoperative follow-up remain important.

## 1. Introduction

The growing success of liver transplantation (LT) in treating end-stage liver disease has established it as the gold standard therapy for many hepatic conditions. Historically, malignancy was considered a contraindication for LT; however, it is now recognized as a potentially curative option for select cancers. The core principle of transplant oncology is to ensure optimal oncologic clearance while addressing the underlying liver disease. Hepatocellular carcinoma (HCC), the most common primary liver malignancy, currently accounts for approximately 20% of liver transplant procedures in the United States [1]. These promising outcomes have expanded LT indications to include other primary hepatic malignancies, such as hemangioendothelioma, hepatoblastoma, and cholangiocarcinoma (CCA) [2]. Although the use of LT for malignancies remains debated, various selection criteria have been established to optimize outcomes and ensure acceptable prognoses.

CCA is the second most common primary liver malignancy, with approximately 20% of cases classified as intrahepatic CCA (iCCA) [3]. In recent years, the incidence of iCCA and iCCA-related mortality has increased across all age and ethnic groups [4,5]. The standard treatment for resectable iCCA involves R0 resection with regional lymphadenectomy. However, only 12–40% of patients are eligible for resection, and recurrence rates remain high at 50–70%, with 5-year overall survival ranging from 25% to 40% [6]. Because of historically poor outcomes, iCCA has been accepted as a contraindication to LT [7]. Nonetheless, recent studies reporting favorable post-transplant outcomes in patients with previously undiagnosed or misdiagnosed iCCA have prompted a re-evaluation of LT as a treatment option [8,9].

iCCA is believed to originate from cholangiocytes and, unlike HCC, is not typically associated with classical risk factors for chronic liver disease, except for primary sclerosing cholangitis (PSC) [10]. A subset of liver neoplasms exhibit both hepatocytic and biliary differentiation, known as “mixed hepatocellular–cholangiocarcinoma (HCC-iCCA)” [11]. In patients with cirrhosis, accurately diagnosing HCC-iCCA preoperatively using tumor markers or cross-sectional imaging remains highly challenging [12]. Similarly, undiagnosed iCCA poses a significant diagnostic difficulty in cirrhotic livers, even when the tumor size is approximately 3 cm [13]. Oncological treatments are advised in pretransplant diagnosed iCCAs, but undiagnosed cases are still debated. For this reason, more information is needed on undiagnosed or misdiagnosed cases. The main problem is preoperative diagnosis and postoperative follow-up. Preoperative markers or postoperative follow-up findings may help achieve a more accurate diagnosis and targeted therapy.

In this study, we evaluated iCCA cases incidentally identified in explant pathology following living donor LT (LDLT).

## 2. Materials and Methods

This retrospective descriptive study was conducted in accordance with ethical standards and approved by the local Ethics Committee (ATADEK-2024/10). Data were collected from all patients who underwent LDLT for presumed HCC at the Organ Transplantation Unit of Acıbadem Atakent Hospital between November 2014 and November 2024. During this period, 985 LDLT procedures were performed, of which 261 cases (26.4%) were diagnosed preoperatively with HCC, and 243 of them were diagnosed as HCC in the explant pathology. At our center, patients diagnosed with iCCA or HCC-iCCA were not considered eligible for LT. All cases in this study involved patients who were either misdiagnosed with HCC or had incidental diagnoses of iCCA or HCC-iCCA on explant pathology. Data analyzed included demographic characteristics (e.g., sex and age, Body Mass Index (BMI)), biochemical markers (e.g., alpha-fetoprotein [AFP, ng/mL], carbohydrate antigen 19-9 [CA 19-9, U/mL], and carcinoembryonic antigen [CEA, ng/mL]), Model for End-Stage Liver Disease (MELD) scores, and preoperative imaging findings. All patients were first evaluated with computed tomography. If suspicious lesions or high AFP levels were present, we also performed magnetic resonance imaging and positron emission tomography/CT (PET-CT). The underlying etiologies of cirrhosis and any pretransplant tumor-directed therapies were also documented.

Liver explants were sectioned at 10 mm intervals, and all grossly abnormal areas were subjected to histological analysis. The diagnosis of iCCA was established based on morphological criteria and confirmed using immunohistochemistry in accordance with the 2019 guidelines of the International Agency for Research on Cancer (International Agency for Research on Cancer, 2019 [14]). The following markers were performed in each case: cytokeratin 7 (Biocare MEDİCAL, Pacheco, CA, USA, OV-TL 12/30), cytokeratin 19 (Thermo, Waltham, MA, USA (A53-5/A2.26)), Hep-PAR (Biocare Medical, Pacheco, CA, USA, OCH1E5), and Arginase-1 (Biocare Medical, Pacheco, CA, USA, EPR6672(B)). The updated Pathologic Evaluation, the College of American Pathologists (CAP) Protocol synoptic reporting format for surgical specimens, covers essential diagnostic elements for staging, including tumor grade, type, margin status, lymph node involvement, vascular and perineural invasion, as well as criteria for differentiating combined hepatocellular carcinoma–cholangiocarcinoma, is used [14,15]. We compared the pathological findings with the preoperative radiological findings. CCA lesions suspected but diagnosed as HCC were termed misdiagnosed. The CCA lesions that were not suspected preoperatively but found after transplantation were termed undiagnosed. Patients were classified using the Goodman classification: type I (separate HCC and iCCA nodules) and type II (combined HCC-iCCA lesions) [16]. In Goodman type I (Collision tumor), HCC and CCA exist as separate, distinct nodules or colliding areas within the same liver. In Goodman type II (Transitional tumor), a single tumor shows intimate intermingling of both HCC and CCA components, with areas of apparent histological transition from one cell type to the other. If only CCA is present, we defined it as a Sole. Preoperative computed tomography (CT) was performed in all transplant cases. In patients with suspected lesions, magnetic resonance imaging (MRI) was performed. Imaging was performed using a [3T MR/SIEMENS, Magnetom Skyra, 1.5T MR/SIEMENS, Magnetom Sola] scanner (SIEMENS, Munich, Germany). Using a T1-weighted three-dimensional (3D) turbo-field-echo sequence, unenhanced, enhanced arterial phase (20–35 s), portal venous phase (60 s), delayed phase (3 min), and 20 min HBP images were obtained for Gd-EOB-DTPA (Primovist; Bayer Healthcare, Berlin, Germany) enhanced MR imaging. We used a respiratory-triggered single-shot echo-planar imaging method with b-values of 0, 50, and 800 s/mm^2^ to obtain DW images. For fat suppression on DW imaging, we used a spectral-attenuated inversion-recovery approach. A monoexponential function with b values of 0 and 800 s/mm^2^ was used to determine the ADC value.

Patients with suspicious HCC lesions were also evaluated with PET-CT to detect metastases. LT was not performed if PET-CT showed extrahepatic or regional lymph node metastases. Patients presenting with a single tumor > 5 cm, more than three tumors (each ≤3 cm), portal vein tumor thrombus involving second-order branches, or AFP levels > 450 ng/mL were recommended for transarterial chemoembolization (TACE). Those treated with TACE underwent LT at least 6 weeks after the procedure. This allowed patients outside Milan criteria to be bridged to transplantation.

Living donors were selected from first- to fourth-degree relatives of recipients or volunteers, provided that they received Ethics Committee approval. The donor evaluation process comprised three stages. The first stage involved complete blood count, viral load testing, and blood type determination. The second stage included triphasic CT and magnetic resonance cholangiopancreatography (MRCP). In the final stage, we performed percutaneous liver biopsy and genetic testing for Factor II and V mutations and methylenetetrahydrofolate reductase variants.

Our immunosuppressive protocol consists of a calcineurin inhibitor (CNI), mycophenolate mofetil (MMF), and corticosteroids. Prednisolone was initiated at 500 mg intravenously during surgery, then tapered from 100 mg to 20 mg over 8 days and further reduced to 5 mg by the second postoperative month, at which point it was discontinued. MMF was added when a reduction in CNI dosing was necessary due to renal impairment. In patients who had HCC out of Milan Criteria, patients who had experienced any cancer after transplantation, and patients with Mis/undiagnosed CCA, CNI was transitioned to Mammalian target of rapamycin inhibitors (mTORi) after 8 weeks, with target trough levels of 6–8 ng/mL maintained until the 6th month. Thereafter, only mTORi was continued, except in patients with Primary Sclerosing Cholangitis (PSC), for whom CNI and mTORi were used in combination. Post-transplant monitoring included serum AFP and CA 19-9 levels. CA19-9 levels were measured unless there was cholangitis or cholestasis. Imaging surveillance comprised ultrasonography every 3 months and magnetic resonance (MRI) every 6 months to detect potential recurrences. We collected data on tumor recurrence, complications, and patient survival from medical records. We evaluated age, sex, BMI, MELD score, CHİLD score, etiology of cirrhosis, waiting time for transplantation, preoperative and postoperative tumor marker levels, postoperative hospital stay, postoperative complications, and patient survival. We also evaluated the number and size of tumor lesions on preoperative imaging and the number, grade, type, characteristics, location, and size of tumor lesions in the pathological results. We compared postoperative explant pathology results with preoperative imaging findings to identify misdiagnoses or undiagnosed malignancies.

All statistical analyses were performed using Number Cruncher Statistical System (NCSS) 2020 (NCSS LLC, Kaysville, UT, USA). We summarized quantitative variables as means, standard deviations, medians, and ranges (minimum–maximum), and presented qualitative variables as frequencies and percentages. We assessed data normality using the Shapiro–Wilk test and box plot visualizations.

For non-normally distributed variables, we used the Mann–Whitney U test to compare two groups, and the Wilcoxon signed-rank test for within-group comparisons. The Kruskal–Wallis test was used to compare three or more groups, followed by the Dunn test for post hoc analysis. Categorical variables were compared using Fisher’s exact test and the Fisher–Freeman–Halton test.

Survival outcomes were analyzed using the Kaplan–Meier method, including overall survival and survival by Goodman classification. Statistical significance was set at *p* < 0.05 at the 95% confidence level.

## 3. Results

### 3.1. Patient Data

The mean age of the patients was 61.67 ± 11.82 years (range, 30–73 years). The cohort comprised 7 females (38.9%) and 11 males (61.1%). The mean body mass index (BMI) was 25.4 ± 4.4 kg/m^2^ (range: 17–33 kg/m^2^). Table 1 summarizes clinical and demographic characteristics. Among 18 patients, there were 42 lesions with suspected HCC. All patients (89.4%), except one (No. 11), were diagnosed with at least one HCC lesion. Preoperative assessments revealed a mean Child–Pugh score of 8.39 ± 1.9 (range: 5–11) and a mean MELD score of 10.4 ± 4.6 (range: 6–21). The average number of tumors identified on preoperative imaging was 2.25 ± 1.3 (range: 1–6), with a mean tumor size of 2.87 ± 1.1 cm (range: 1–5 cm). All patients had underlying cirrhosis of various etiologies: hepatitis B (50%), cryptogenic cirrhosis (16.7%), nonalcoholic steatohepatitis (11.1%), PSC (11.1%), hepatitis C (5.6%), and alcoholic cirrhosis (5.6%). The mean waiting time from indication for transplantation to LT was 0.94 ± 0.55 years. All biliary anastomoses were duct-to-duct.

### 3.2. Pathological Findings

A total of 985 LDLT procedures were performed. Among them, 261 patients with suspected HCC underwent LDLT. Among patients who underwent LDLT because of suspected HCC (261), 18 patients (1.8%) had CCA on explant pathology. Preoperative imaging showed 42 lesions, whereas pathology identified 55. Among 8 patients with Goodman type 2 lesions, 9 new (undiagnosed) and 11 misdiagnosed lesions were detected. In Goodman type 1, both HCC and iCCA are present. In Goodman type 1, 2 lesions were undiagnosed, and 3 were misdiagnosed. In the sole iCCA group, there were 11 lesions (misdiagnosed) and 2 undiagnosed lesions. Two lesions thought to be HCC were found to be dysplastic nodules on pathological evaluation. The total number of misdiagnosed and undiagnosed lesions was 25 and 13, respectively. The total number of iCCA lesions was 38. Table 2 shows the distribution of undiagnosed and misdiagnosed lesions.

Among the patients recommended for TACE, three were unable to undergo the procedure for various reasons and were therefore promptly taken to surgery. Seven patients (38.9%) received TACE treatment. One patient (case 17), whose lesion was near the right hepatic vein, received external beam radiotherapy (RT). The mean preoperative levels of CA 19-9, CEA, and AFP were 41.34 ± 39.2 U/mL, 4 ± 5.2 ng/mL, and 62.55 ± 91.5 ng/mL, respectively. Following TACE or Radiotherapy (RT), AFP levels decreased to <450 ng/mL.

Table 2 presents the pathological findings. Based on the Goodman classification, five patients (27%) had type I lesions, eight (44.4%) had type II lesions, and five (27.8%) had CCA lesions alone. One case exhibited both a Goodman type II lesion and a sole HCC lesion. The mean number of HCC and CCA lesions was 2.83 ± 0.75 and 2.11 ± 2.2, respectively. The average diameter of HCC tumors was 2.12 ± 0.8 cm, whereas that of CCA tumors was 3.06 ± 1.64 cm. Among HCC lesions, 4 (57.1%) were well-differentiated, 2 (28.6%) were moderately differentiated, and 1 (14.3%) was poorly differentiated. For CCA lesions, 1 (5.6%) was well-differentiated, 13 (72.2%) were moderately differentiated, and 4 (22.2%) were poorly differentiated. All tumors treated with TACE or RT showed evidence of necrosis. Vascular invasion was present in 50% of lesions, and perineural invasion in 33%. Tumor localization included the right lobe in 11 patients (61.1%), left lobe in 2 patients (11.1%), hilar region in 2 patients (11.1%), and both lobes in 3 patients (16.7%).

### 3.3. Outcomes

Table 3 presents patient outcomes after LT.

The mean hospital stay after LT was 13.11 ± 4.25 days (range, 7–22 days) after transplantation. Mild acute rejection occurred in six patients (33%), all of whom were successfully managed with high-dose corticosteroids. Tumor recurrence was observed in six patients (33.3%), with a mean disease-free survival of 10.67 ± 5.7 months. The average follow-up duration was 32.4 ± 29 months (range, 9–113 months).

Biliary complications occurred in 8 patients (44%), a significantly higher rate than in high-volume centers [17]. The bile anastomosis types were duct-choledoch (11 cases), two united bile ducts-choledoch (3 cases), two ducts to choledoch and cystic duct (1 case), hepaticojejunostomy (2 cases), 3 ducts to cystic duct, right bile duct, and left bile duct (1 case). The complications occurred in two united bile ducts (choledoch) (3 cases), two ducts to the choledoch and the cystic duct (1 case), 3 ducts to the cystic duct, the right bile duct, and the left bile duct (1 case), and duct-choledoch (3 cases). One patient succumbed to cholangitis and sepsis secondary to a biliary complication. Patients with recurrence received chemotherapy in external oncology centers. Two patients died due to COVID-19 during the pandemic period. Another patient developed advanced-stage squamous cell carcinoma of the lung 61 months post-transplant. Among our patients, 4 of them experienced mild acute cellular rejection, which was diagnosed with liver biopsy. They were treated with high-dose steroids (250 mg) for 3 days. None experienced tumor recurrence at follow-up.

Among the six patients with recurrence, the average time to recurrence was 10.6 months (range, 4–20 months), with recurrence sites evenly distributed among the lymph nodes (33%), lungs (33%), and liver (33%). No significant differences were found between patients with and without recurrence in terms of MELD score, tumor size, tumor count, or cirrhosis etiology (*p* = 0.55, *p* = 0.87, *p* = 0.56, and *p* = 0.67, respectively). As expected, both preoperative and postoperative CA 19-9 levels were significantly higher in patients with recurrence than in those without recurrence (preoperative: 74.88 ± 46 vs. 24.5 ± 21 U/mL, *p* = 0.01; postoperative: 133.83 ± 115 vs. 11.9 ± 12.9 U/mL, *p* = 0.01). No significant differences were observed in AFP levels between the two groups, either preoperatively (*p* = 0.55) or postoperatively (*p* = 0.08). Although AFP levels remained unchanged post-transplant in patients with recurrence (*p* = 0.9), they decreased significantly in non-recurrent cases (preoperative: 10.1 ng/mL; postoperative: 1.6 ng/mL, *p* = 0.02). No significant differences were found in relation to tumor necrosis, vascular invasion, perineural invasion, or tumor location between the two groups (*p* = 0.6, *p* = 1, *p* = 1, and *p* = 0.37, respectively). Recurrence rates by Goodman classification were as follows: type I, 3 cases (60%); type II, 2 cases (25%); and sole CCA, 1 case (20%). The mean disease-free survival times were 14.00 ± 5.57 months for type I, 8.00 ± 5.66 months for type II, and 6.00 ± 0.00 months for sole CCA.

**Figure 1:**
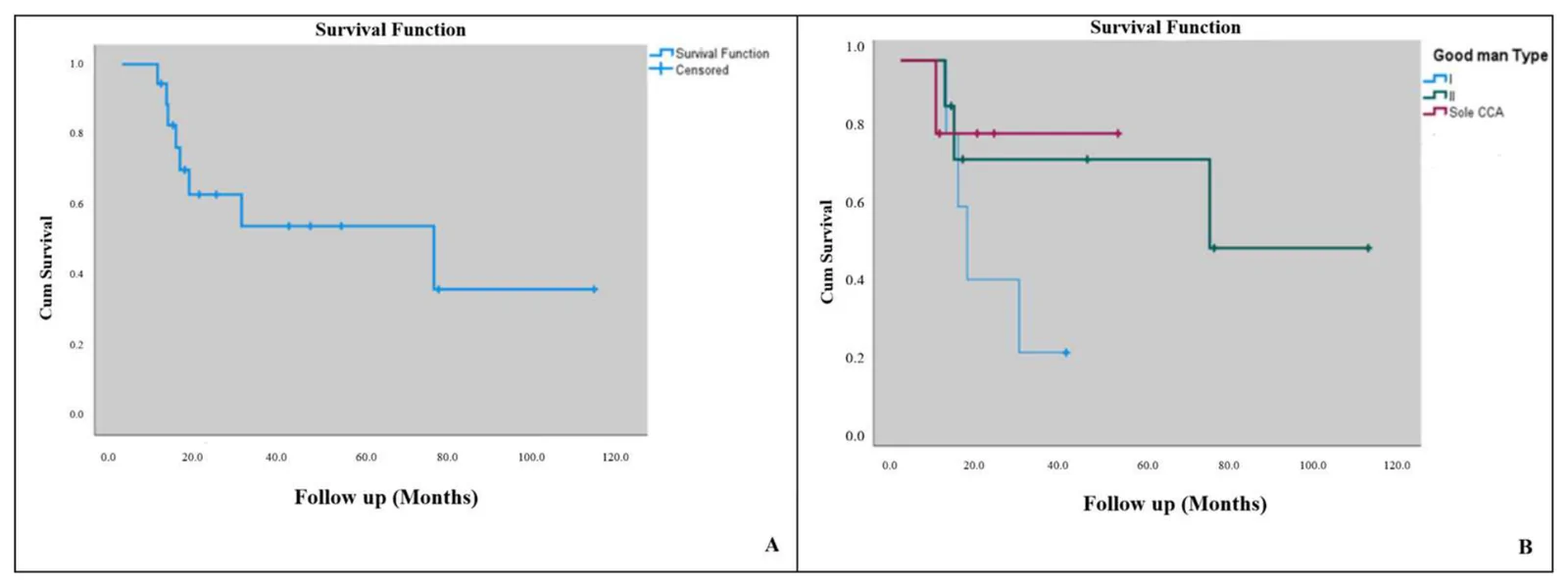
Survival graphs. (**A**) General survival analysis. (**B**) Survival analysis according to the Goodman classification (*p* = 0.31).

In the survival analysis, the mean survival time was 61.6 ± 12 months (Figure 1). The mean survival durations for the Goodman type I, type II, and sole CCA groups were 22.03 ± 4.8, 76.8 ± 16, and 44 ± 7.9 months, respectively. The proportions of surviving patients in each group were 0.20 (type I), 0.6 (type II), and 0.8 (sole CCA). Although the Goodman type I group exhibited a trend toward lower survival, the difference was not statistically significant (*p* = 0.316). Across all cases, 1-year survival was 88%, and 5-year survival was 55%. Among causes of death, four patients died from CCA recurrence, whereas the remaining deaths were due to sepsis, COVID-19, amyotrophic lateral sclerosis, and lung squamous cell carcinoma. mTORi treatment was continued in 14 cases. mTORi was switched to a steroid in two cases with cholangitis-related sepsis. mTORi was switched to CNI in two patients due to severe oral aphthous lesions.

### 3.4. Comparative Analysis

Comparative analysis showed no significant differences among the Goodman classification groups in sex, age, or BMI (*p* = 0.609, *p* = 0.182, and *p* = 0.181, respectively). Similarly, MELD scores and tumor counts did not differ significantly across groups (*p* = 0.44 and *p* = 0.07, respectively). However, tumor size was significantly larger in the sole CCA group compared to the Goodman type I and II groups (*p* = 0.016). There were no statistically significant differences in preoperative versus postoperative CA 19-9 levels among the Goodman types (*p* = 0.5 for type I, *p* = 0.48 for type II, and *p* = 0.68 for sole CCA). AFP levels significantly decreased in the Goodman type II group postoperatively (*p* = 0.012). In contrast, no significant changes were noted in the Goodman type I or sole CCA groups (*p* = 0.5 and *p* = 0.5, respectively). No significant intergroup differences were found regarding the number of CCA tumors, average CCA tumor diameter, tumor grade, vascular invasion, or perineural invasion (*p* = 0.29, *p* = 0.2, *p* = 0.4, *p* = 0.4, and *p* = 0.3, respectively). Furthermore, recurrence rates, disease-free survival, and mortality did not significantly differ across the Goodman groups (*p* = 0.45, *p* = 0.3, and *p* = 0.2, respectively).

## 4. Discussion

This retrospective descriptive study describes incidental and misdiagnosed CCA cases in LDLT. Previous studies on undiagnosed/misdiagnosed CCA have had limited patient numbers, making statistical comparisons difficult and reducing reliability [7,8,9,13]. Chronic biliary diseases, such as PSC, are the best-known predisposing factors for CCA. However, cirrhosis has recently emerged as a potential predisposing condition [18]. Several studies have indicated that cirrhosis secondary to alcohol use and hepatitis C significantly increases the risk of CCA, with a reported odds ratio of 27.2 in the U.S. population [19]. Similarly, a French study found that CCA was most frequently observed in patients who underwent LT due to alcoholic cirrhosis [13]. However, a prospective cohort study from Japan identified a relatively high incidence of CCA among patients with HCV-related cirrhosis [20]. In our study, we did not identify a dominant underlying etiology, which may reflect the global epidemiological diversity of cirrhosis etiologies [21]. A systematic review reported that the overall incidence of misdiagnosed or undiagnosed CCA in LT recipients was approximately 0.7%, a rate higher than that of de novo CCA and suggestive of cirrhosis as a contributing risk factor [22]. Vallin et al. reported a 1% incidence of CCA in cirrhotic patients and a 3% incidence among all explanted tumors [13]. Notably, their study included only intrahepatic CCA cases and excluded mixed tumors. In another report, the incidence of mixed HCC-iCCA was 0.7% [23]. In comparison, our study found a substantially higher incidence, including both intrahepatic CCA and mixed tumor types (HCC-iCCA and combined HCC and CCA lesions). Liver tumors typically arise in the right hepatic lobe. Although some literature suggests that CCA is more commonly located in the left lobe, other studies have shown a predominance of right-lobe involvement, particularly in mixed tumors [11,24]. Consistent with these findings, 61% of tumors in our cohort were located in the right lobe. Furthermore, although none of the tumors were centered at the main duct bifurcation, two cases were found near the hepatic hilum.

Recent studies have reported that the mean age of patients with misdiagnosed or undiagnosed iCCA or mixed HCC-iCCA undergoing LT ranges between 58 and 61 years [22]. Similarly, the mean age of the patients in our study was 61 years. All patients under 52 years of age in our study were diagnosed with PSC. Consistent with prior reports, most patients were males [22].

Accurate preoperative diagnosis of CCA in patients with liver cirrhosis remains challenging due to nonspecific clinical presentation and imaging features that may resemble HCC or benign nodules. In previous studies, MRI of histologically confirmed CCA in cirrhotic livers identified progressive contrast enhancement and absence of washout as suggestive features of CCA [25]. However, approximately 20% of lesions lacked these characteristics. Moreover, CCA arising from a cirrhotic liver may be enclosed by fibrotic pseudocapsules, often presenting as capsular retraction [26]. Biliary ductal dilation, a typical finding in non-cirrhotic livers, may not be evident in cirrhotic cases. Despite established radiological hallmarks, multiple reports indicate that distinguishing CCA from HCC in cirrhotic livers remains difficult [12,27]; diagnostic accuracy further declines for non-enhancing lesions and those smaller than 2 cm. Prior studies describing imaging features of CCA typically involved lesions averaging 3 cm, with characteristic features seen predominantly in lesions larger than 2 cm [25,26]. In our study, 12 of 28 lesions were <2 cm, and none exhibited the radiological features of CCA. This finding indicates that despite tumors being detectable on preoperative imaging, differentiation from HCC may be difficult. A limitation is that 22% of cases were not evaluated with MRI before transplantation. This underscores the importance of routine MRI evaluation in cirrhotic patients being assessed for HCC to improve diagnostic accuracy. According to a recent consensus report, CT and MRI are recommended for evaluating liver lesions. Although biopsy is not recommended for characteristic HCC on radiological findings, it is recommended for lesions with atypical features [28].

Patients with CCA or HCC who undergo LT are typically cirrhotic and tend to have smaller tumors, whereas patients undergoing surgical resection are generally older and non-cirrhotic. These differences make direct comparisons between the two groups challenging. For cirrhotic patients diagnosed with CCA, treatment options such as chemotherapy or surgical resection can be considered. In a study by Lunsford et al., 12 patients with nonmetastatic, unresectable CCA (>2 cm) received neoadjuvant gemcitabine and platinum-based chemotherapy, followed by LT, with only three cases of recurrence reported [29]. These findings support the benefit of neoadjuvant therapy in enhancing LT outcomes. In the recent consensus report, locoregional treatment is recommended to bridge the gap [28]. In our cohort, none of the patients were diagnosed with CCA preoperatively. In our country, due to a shortage of deceased donor organs, CCA is not an accepted indication for cadaveric LT. Therefore, LDLT remains the only viable option in such settings. All of our patients underwent LDLT within a year of transplant indication, highlighting LDLT as a practical solution in countries with limited organ availability. Although most studies in the literature focus on cadaveric transplantation, our findings contribute valuable data on LDLT for CCA, reinforcing that cirrhotic patients with CCA are not contraindicated for LDLT [30]. In a recent consensus report, LDLT has an advantage over deceased donor liver transplantation (DDLT) because of the time advantage after bridging therapies [28].

Previous studies have suggested that elevated CA 19-9 levels indicate CCA in patients with atypical liver lesions [22]. However, in our cohort, only 44% of the patients had elevated CA 19-9 levels, whereas 55% exhibited elevated AFP levels. Earlier studies have reported elevated CA 19-9 levels in 63% of cases, which may be attributable to the predominance of Goodman type I tumors (44%) in our series. Notably, patients who experienced recurrence had significantly higher preoperative CA 19-9 levels than non-recurrent cases, suggesting a link between elevated CA 19-9 and tumor aggressiveness. In recent studies, only radiological follow-up is recommended after liver transplantation for CCA [28,31].

Survival and recurrence rates following LT for CCA vary widely in the literature. In studies by Schwenk and Facciuto, outcomes for patients with combined HCC-CCA were comparable to those for patients with HCC alone [8,32]. In particular, Schwenk reported 1- and 5-year survival rates of 87.5% and 75%, respectively, particularly in cases where tumors were <2 cm. Similar to our study, the small sample size and heterogeneity limit the ability to draw a definitive conclusion. These favorable outcomes are encouraging; however, other studies have reported 5-year survival rates ranging from 16% to 51%, underscoring heterogeneity in outcomes [8,12,26]. The poor prognosis often associated with CCA is believed to result from its inherently aggressive biology. In a study by Sapisochin et al., patients with Goodman type I lesions had better outcomes than those with Goodman type II lesions. However, Facciuto et al. have reported conflicting data [26,32]. In our preliminary results, we observed higher disease-free survival in Goodman type I cases, while overall survival was longer in Goodman type II cases. However, this discrepancy may reflect higher non-tumor-related mortality (e.g., COVID-19, cholangitis) in the type I group. The worst prognosis observed in Goodman type II may be linked to tumor origin from pluripotent hepatic progenitor cells. The small number of cases prevents us from drawing a definitive conclusion. The recurrence rate of CCA cases after LT ranged between 28.6% and 80% in several studies [26,31,32]. In the study by Vallin et al., the tumor recurrence rate was 50%, the 1-year survival rate was 80%, and the 5-year survival rate was 24% [13]. In CCA surgery, lymph node assessment is very important, but in the transplantation era, we do not perform it during the recipient hepatectomy. Our study also confirms that the most important metastatic site in CCA is the hilar lymph nodes. In recent consensus statements, LDLT has better overall and disease-free survival outcomes than DDLT, especially in regions with long waiting times (>3 month) or where LDLT is the predominant type of LT [28]. For patients with preoperatively diagnosed CCA, preoperative bridging therapy or exclusion for transplantation can be performed. But for undiagnosed cases, postoperative follow-up and treatment are gaining importance. Recommended protocols involve reducing calcineurin inhibitor (CNI) exposure by adding mammalian target of rapamycin inhibitors (mTOR). In recent consensus, defining intermediate- and high-risk patients is prioritized for surveillance; our findings might help define patient risk [28]. Steroid use for mild rejections did not result in recurrence. This is probably because of low steroid dosage.

Several poor prognostic factors for tumor recurrence have been cited in the literature, including tumor size >2 cm, exceeding Milan criteria, poor differentiation, multicentric tumors, vascular and perineural invasion, and elevated CA 19-9 [33]. In our small-sample study, elevated preoperative CA 19-9 levels were associated with recurrence. Although this is a preliminary finding, larger studies are needed to confirm this hypothesis. Although we used several comparative tests in our small-sample study, we did not identify a clear risk factor. This is similar to previous studies, where small sample size is again a limiting factor [31]. Once large datasets on preoperative imaging of CCA are available, artificial intelligence will help in future studies.

## 5. Conclusions

Diagnostic challenges, delisting upon diagnosis in cirrhotic patients, biological heterogeneity, and lack of standardized postoperative adjuvant therapy continue to complicate the management of iCCA in the transplant setting. Although transplant oncology is gaining prominence, preoperative evaluation and postoperative follow-up remain important.

## Figures and Tables

**Table 1:** Clinical characteristics of patients and tumors included in the study.

Cases	Age (Years)	BMI (kg/m^2^)	Etiology of Cirrhosis	Sex (Male/Female)	MELD Score	Child Pugh Score	Preop Ca19-9 (U/mL)	Preop CEA (ng/mL)	Preop AFP (ng/mL)	Pretransplant Treatment	Number of Tumors in Preoperative Radiological Evaluation	Radiological Size of Tumors (cm)
1	57	24.09	HBV	Male	10	A-5	**47.00**	2.4	**106**	−	3	3–1.5–1.5
2	69	24.77	HBV	Male	13	A-6	3.53	1.7	**2240–54** (After TACE)	TACE	1	3.00
3	64	19.72	HCV	Male	10	B-7	**107.00**	2.9	**36.0**	TACE	2	4.2–1.5
4	57	21.88	HBV	Female	6	A-6	20.70	2.9	**66.6**	−	4	2.1–1–1–1
5	66	28.89	HBV	Female	15	B-7	**47.50**	3.6	2.8	−	1	1.1
6	73	25.91	Cryptogenic	Female	21	C-11	**104.00**	4.3	**43.9**	−	2	1.1–0.7
7	60	32.95	Alcohol	Male	10	B-8	**38.90**	**5.2**	**14.1**	TACE	6	3–1.7–1.5
8	69	18.36	Cryptogenic	Female	6	C-10	1.52	5	**78.9**	TACE	2	4–0.6
9	71	22.92	HBV	Male	14	C-11	23.90	1	**119**	−	2	4–0.8
10	30	16.94	PSC	Female	7	C-11	**136.00**	24	1.4	−	1	5.00
11	37	27.76	PSC	Male	7	B-9	24.50	1	1.4	−	0	0.00
12	69	23.32	HBV	Female	6	B-8	22.30	2.7	6.1	TACE	3	3.2–1.7–1.2
13	67	30.04	Cryptogenic	Male	6	B-8	18.60	5	2.55	TACE	3	3.0–1
14	73	32.32	HBV	Male	6	B-8	**77.70**	5	6.06	−	1	2.7
15	62	28.41	HBV	Male	6	B-8	12.00	1	1.01	−	3	2–1.5–1
16	69	26.49	NASH	Male	14	C-10	**33.00**	2.4	**261**	TACE	1	1.50
17	65	26.47	HBV	Male	18	C-11	22.00	1	**1300–320** (After RT)	RT	1	3
18	52	25.80	NASH	Female	12	B-7	4	1	5	−	6	−

HBV: Hepatitis B Virus; HCV: Hepatitis C Virus; PSC: Primary Sclerosing Cholangitis; MELD: Model for End-Stage Liver Disease; TACE: Trans-Arterial Chemo-Embolisation; RT: Radiotherapy; NASH: Non-Alcoholic Steatohepatitis; High Values given in bold. Normal Ranges for CA 19-9: 0–37 U/mL; CEA: 0–4.6 ng/mL; AFP: 0–9.9 ng/mL.

**Table 2:** Pathological findings of Tumors.

Cases	Good Man	Tumor Types	# of HCC Tumors	Size of HCC (cm)	Grade of HCC	# of CCA Tumors	Size of CCA (cm)	Tumor Grades of CCA	IHC Staining Positivity	Tumor Necrosis	Diagnostic Error	Vascular Perineural İnvasion	Localization of CCA
1	I	HCC + CCA	2	2.5 + 1.3	Well	1	1.20	Well	CK19, CK7 and Arg, HSA	−	Mis	V: −/P: +	R
2	II	Mix				1	2	Moderate	CK19, CK20, CK7, MOC-31, CEA-P	+	Mis	V: −/P: −	L
3	I	HCC + CCA	4	2 + 2 + 2 + 1.5	Moderate	1	3.5	Moderate	CK-7, CK20, Arg, HSA	+	Mis + Un	V: +/P: −	R
4	II	Mix				7	Range: 0.6–21	Moderate	CK19, CK20, CK7, MOC-31	+	Mis + Un	V: +/P: −	R + L
5	II	Mix			Moderate	3	2.1–1.5–1	Moderate	CK7, CK19	−	Mis + Un	V: +/P: −	L
6	II	Mix				5	Range 0.3–4.3	Moderate	CK7, HSA, CK19	+	Mis + Un	V: +/P: −	R
7	I	HCC + CCA	3	1.1 + 0.9 + 0.5	Poor	1	2.5	Moderate	CK7, CK19, HSA, Arg	+	Mis	V: −/P: −	R
8	Sole	CCA				2	3 + 1.5	Moderate	CK7, CK19	+	Mis	V: −/P: −	R + L
9	Sole	CCA				1	4	Moderate	CK7, CK19	−	Mis	V: −/P: −	R
10	Sole	CCA				1	4	Moderate	CK7, CK19	−	Mis	V: +/P: +	H
11	Sole	CCA				1	2	Moderate	CK7, CK19	−	Un	V: +/P: +	H
12	I	HCC + CCA	3	2.7–1.4–0.9	Well	1	4	Moderate	CK7, CK19, and Arg, HSA	+	Mis	V: −/P: −	R
13	II	Mix + CCA	3	1.3–1–1–0.9	Well	1	2.9	Moderate	CK7, CK19, Arg, HSA	+	Mis + Un	V: +/P: +	R
14	II	Mix				1	1.7	Poor	CK7, CK19, Arg, HSA	−	Mis	V: −/P: −	R
15	I	HCC + CCA	2	3.1–1.7	Well	1	1.3	Moderate	CK7, CK19, HSA	−	Mis	V: −/P: +	R
16	II	Mix				1	2	Poor	CK7, CK19, and Arg, HSA	+	Mis	V: −/P: −	R
17	II	Mix				1	3.50	Poor	CK7, CK19, and Arg, HSA	+	Mis	V: +/P: −	R
18	Sole	CCA				8	Range 0.2–2	Poor	CK7, CK19	−	Un	V: +/P: +	R + L

IHC: Immunohistochemical; CCA: Cholangiocarcinoma; HCC: Hepatocellular Carcinoma; Mis: Misdiagnosed; Un: Undiagnosed; R: Right; L: Left; H: Near Hilus; Good man Type I: Separate nodules of HCC and iCCA; Goodman type II: Combined HCC-iCCA lesions, Vascular (V: +/−)/Perineural İnvasion (P: +/−), Mix: Mix Type, Sole: Sole CCA, #: Number. +: Exist, −: None.

**Table 3:** Outcomes of patients after liver transplantation.

Cases	Hospital Stay (Day)	Complication	Rejection	Postop AFP (ng/mL)	Postop Ca 19-9 (U/mL)	Tumor Recurrence	Recurrence Time (Month)	Mortality	Mortality Reason	Mortality Time (Month)
1	7	Biliary Stricture-ERCP	−	1.2	154	+	9	+	Cholangitis-Sepsis	14
2	13	−	−	1.2	24	−	−	+	COVID-İleus-sepsis	12
3	10	Bile leakage-ERCP	−	25	50	+	13	+	CCA	15
4	10	−	−	4	2.8	−	−	−	−	−
5	12	−	−	1.2	2.6	−	−	+	Amyotrophic Lateral Sclerosis	75
6	9	−	−	4.2	270	+	4	+	CCA	8
7	13	Bile leakage—ERCP	−	4.2	45	−	−	−	−	
8	15	Bile leakage-ERCP	−	5	2	−	−	−	−	−
9	13	Bile Leakage- ERCP	−	1.2	23	−	−	−	−	−
10	17	−	−	639	270	+	6	+	CCA	10
11	22	Bile stricture-ERCP-PTC	+ (steroid)	1.3	5	−	−	−	−	−
12	7	Bleeding—Laparotomy	−	179	36	+	20	+	CCA	30
13	12	−	−	1.2	12	−	−	−	−	−
14	13	Bile stricture-ERCP	+ (steroid)	4.7	5	−	−	−	−	−
15	21	Bile stricture-ERCP	−	0.6	6.4	−	−	+	Squamous cell carcinoma of lung	47
16	18	Bile stricture-ERCP	−	1.6	23	+	12	−	CCA	−
17	10	Bile stricture-ERCP	+ (steroid)	1.8	13	−	−	−	−	−
18	14	−	+ (steroid)	1.8	2	−	−	−	−	−

ERCP: Endoscopic retrograde cholangiopancreatography; PTC: Percutaneous Transhepatic Cholangiography; CCA: Cholangiocarcinoma. +: Exist, −: None.

## Data Availability

The data supporting the findings of this study are not publicly available due to sensitivity, but can be obtained from the authors upon reasonable request.

## Abbreviations

AFP	Alpha-fetoprotein
BMI	Body Mass Index
CA 19-9	Carbohydrate antigen 19-9
CEA	Carcinoembryonic antigen
LT	Liver transplantation
CCA	Cholangiocarcinoma
CNI	Calcineurin inhibitor
DDLT	Deceased donor liver transplantation
HCC	Hepatocellular carcinoma
HCC-iCCA	Mixed hepatocellular-cholangiocarcinoma
iCCA	Intrahepatic CCA
LDLT	Living donor liver transplantation
MELD	Model for End-Stage Liver Disease
MMF	Mycophenolate mofetil
MRCP	Magnetic resonance cholangiopancreatography
MRI	Magnetic resonance
mTORi	Mammalian target of rapamycin inhibitors
PET CT	Positron emission tomography/CT
PSC	Primary Sclerosing Cholangitis
RT	Radiotherapy
TACE	Transarterial chemoembolization
